# Canine intraocular pressure dynamics during mild-pain ophthalmic procedures in three premedication protocols

**DOI:** 10.14202/vetworld.2025.573-581

**Published:** 2025-03-09

**Authors:** Laura Voiko, Armands Vekšins, Diāna Birnere, Liga Kovalcuka

**Affiliations:** Clinical Institute, Faculty of Veterinary Medicine, Latvia University of Life Sciences and Technologies, Jelgava, Latvia

**Keywords:** acepromazine, butorphanol, dog, intraocular pressure, medetomidine, premedication

## Abstract

**Background and Aim:**

Maintaining intraocular pressure (IOP) stability during ophthalmic procedures is essential to ensuring surgical success and reducing complications related to IOP fluctuations. This study aimed to evaluate IOP dynamics in dogs undergoing mild-pain ophthalmic procedures under three different premedication protocols: butorphanol alone (B), butorphanol with medetomidine (BM), and butorphanol with acepromazine (BA).

**Materials and Methods:**

Thirty clinically healthy client-owned dogs of various breeds (19 males, 11 females, aged 4 months–11 years, weight 7.8–79 kg) were randomized into three groups. IOP was measured at multiple perioperative time points using rebound tonometry: premedication (T0), 5 (T5), and 10 (T10) min after premedication, after intubation (Tint), before surgery (Tbsur), post-surgery (Tasur), during extubation (Text), and before discharge (Tlea). Statistical analyses, including t-tests and Pearson correlation, were performed to assess differences in IOP within and between groups.

**Results:**

Significant changes in IOP were observed at different time points across groups. The B group showed a significant IOP increase between T0 (15.55 ± 3.50 mmHg) and Tint (19.3 ± 4.19 mmHg) (p < 0.05). In the BM group, IOP increased from T0 (15.9 ± 2.77 mmHg) to Tint (19.15 ± 4.52 mmHg) and decreased significantly postoperatively at Tasur (13.5 ± 3.50 mmHg). The BA group exhibited significant IOP reductions from T0 (20.35 ± 2.78 mmHg) to Tbsur (16.45 ± 3.97 mmHg) and Tlea (17.15 ± 4.22 mmHg). No correlation was found between IOP and sex, breed, or age.

**Conclusion:**

IOP remained within normal ranges throughout the perioperative period in all groups. Acepromazine, in combination with butorphanol, was the most effective in attenuating the IOP increase caused by intubation, suggesting its potential advantage in patients at risk of corneal perforation. Clinically, premedication selection should prioritize minimal IOP fluctuation to enhance surgical outcomes.

## INTRODUCTION

Balanced general anesthesia is an important component of any surgery, including ophthalmic surgery, to eliminate pain and tranquilize the animal for a safe procedure. In addition to hypnosis, controlling pain levels, maintaining the central eye position for corneal and intraocular procedures, and achieving stable intraocular pressure (IOP) are essential factors in veterinary ophthalmology [1, 2]. Controlling IOP before, during, and after ophthalmic surgery is crucial for successful outcomes [3]. Surgical procedures can be characterized as operations inducing mild pain; for example, removal of distichiasis, minor operations on the conjunctiva and eyelids, and reposition of a prolapsed nictitans gland; moderate pain – treatment of severe entropion, repair of acute and deep corneal ulcers, and severe pain – enucleation and major operations on the cornea, and eyeball perforation or evisceration procedures [4, 5]. Therefore, the selection of pre-anesthetic and anesthetic drugs for particular operations is important [5]. An increase in IOP can lead to serious complications that impair vision. These include deep corneal ulcer perforation, potentially leading to eyeball loss, hemorrhage, retinal detachment, and a sudden perioperative increase in IOP after cataract surgery, which is likely to cause acute glaucoma [6, 7]. In dogs, various pre-anesthetic drugs such as butorphanol, medetomidine, and acepromazine (ACP) are used, either alone or in combination. Although the general effects of anesthetic agents have been extensively documented, the impact of varying combinations and dosages of these agents on IOP remains relatively underexplored and subject to debate.

Butorphanol tartrate is a kappa opioid receptor agonist and m-opioid receptor antagonist. Despite its weak analgesic properties [8], it can achieve mild-to-moderate short-term sedation in dogs with minimal side effects [9–11]. An increase in IOP has been reported even at 45 min after intramuscular (IM) injection, but it is tolerable in ophthalmologically healthy dogs [11, 12]. In contrast, no effect on IOP has been reported [13]. Medetomidine is an alpha-2-adrenergic agonist with dose-dependent sedative, analgesic, and muscle relaxant effects in animals [14, 15], with the potential to lower blood pressure and heart rate. Researchers have observed no effect of medetomidine on IOP after intravenous (IV) injection of 1500 μg/m^2^ body surface area or a significant decrease in IOP following IM injection of 10 mg/kg [16, 17]. Furthermore, concomitant IV administration of medetomidine and butorphanol increased IOP after 10 min but not after 30 or 40 min [18]. Studies by Norman *et al*. [19] and Artigas *et al*. [20] demonstrated a reduction in IOP with medetomidine and dexmedetomidine use, whereas five other reports found no effect [13, 17, 21–23]. Conversely, the combination of medetomidine and dexmedetomidine increased IOP when combined with butorphanol [18].

Finally, ACP maleate is a phenothiazine with a sedative effect in dogs that reduce IOP as early as 30 min after IM injection [24]. When administered intravenously, 0.015 mg/kg ACP increased IOP [23]; however, another study by Mrazova *et al*. [13] reported that a dose of 0.02 mg/kg had no effect. Furthermore, the combination of IM medetomidine and ACP resulted in a non-significant reduction in IOP in dogs [16].

Drugs used as premedication in a general anesthesia protocol should be selected according to the type of surgery and individual health status of the animal. Therefore, our objective was to investigate the effects of different pre-anesthetic drugs and their combinations (butorphanol tartrate alone, butorphanol and medetomidine hydrochloride, and butorphanol and ACP maleate) on IOP in the perioperative period. Previous studies by Lerche [2], and Pierce-Tomlin *et al*. [3] investigated the effects of butorphanol, medetomidine, and ACP on IOP in dogs were documented.

Therefore, this study evaluated the IOP of different doses of an IM pre-anesthetic drug combination following IV midazolam/propofol for anesthetic induction in healthy dogs. To our knowledge, no previous study has investigated the effects of premedication doses we used to treat IOP. We hypothesized that IOP would remain within the normal range throughout the perioperative period in healthy dogs.

## MATERIALS AND METHODS

### Ethical approval

This study received ethical approval from the Animal Welfare and Protection Ethics Council of the Latvia University of Life Sciences and Technologies (LLU_Dzaep_2022-5, October 11, 2022). Each procedure was done in accordance with the *in vivo* study guidelines (ARRIVE).

### Study period and location

The study was conducted from February to December 2023 at the University Veterinary Clinic in Jelgava.

### Animals

A prospective, randomized, and blinded clinical study was conducted on 30 dogs of different breeds (Table 1), including 19 males and 11 females aged 4 months–11 years with an average weight of 26.6 ± 19.35 kg (range, 7.8–79 kg).

**Table 1:** Dog breeds.

Breed	Number
Cane Corso	6
Mongrel	4
French Bulldog	4
Tibetan Mastiff	2
Pug	2
American Staffordshire Terrier	2
Central Asian Shepherd Dog	2
ShihTzu	1
Jack Russell Terrier	1
Bavarian Mountain Hound	1
Chow Chow	1
American Pit Bull Terrier	1
Doberman	1
American Cocker Spaniel	1
Beagle	1

Dogs were admitted for ophthalmic surgery. Before surgery, routine clinical and ophthalmologic examinations were conducted. All dogs included in the study were clinically healthy. The health status of each animal was assessed using hematological and biochemical tests. In addition, dogs with Class I or II health status (American Society of Anesthesiologists [ASA]) were eligible to participate in the study [25]. Animals younger than 4 months or older than 11 years, those receiving concurrent medications, those with concurrent diseases, or those classified as ASA Class III or higher were excluded from the study. In addition, canine behavior was evaluated using the modified pre-anesthesia and recovery behavior scoring criteria. The study did not include dogs with a score of 4, indicating aggressiveness or uncooperativeness [26].

The ophthalmic examination included direct ophthalmoscopy (Keeler Practitioner, Windsor, UK), monocular ophthalmoscopy with the PanOptic ophthalmoscope (Welch Allyn, Windsor, UK), slit-lamp biomicroscopy (Kowa SL15, Nagoya, Aichi, Japan), and D-dog-calibrated rebound tonometry (TonoVet®, Tiolat Ltd., Vantaa, Finland). Dogs undergoing the following procedures characterized by mild surgical pain were included in the study: third eyelid gland prolapse (n = 12), entropium (n = 9), ectopic cilia (n = 3), minor eyelid tumors (n = 4), and third eyelid cartilage eversion (n = 2). We recorded whether the surgery was unilateral or bilateral and the level of discomfort or pain shown by the animal.

Although this study is clinical in nature, animals were recruited, clinically examined, and anesthetized under highly uniform conditions. In addition, a standardized protocol was employed for all IOP measurement techniques.

### Anesthetic management

To ensure that the duration and quality of individual procedures were approximately the same, a single approved surgical team performed all operations in the morning. All animals were denied food but not water for ≤8 h before being anesthetized. Dogs were randomly divided into three groups (n = 10 each). The three groups received the following analgesic premedication protocols: (1) group butorphanol alone (B): butorphanol 0.2 mg/kg, 10 mg/mL (Butomidor, Richter Pharma, Wels, Austria); (2) group butorphanol with medetomidine (BM): butorphanol 0.2 mg/kg with medetomidine 0.005 mg/kg, 1 mg/mL (Domitor, OrionPharma, Espoo, Finland); and (3) group butorphanol with acepromazine (BA): butorphanol 0.2 mg/kg with ACP 0.02 mg/kg, 10 mg/mL (Neurotranq, Alfasan, Woerden, Netherlands). Upon arrival at the clinic, each dog’s behavior was assessed using a descriptive scale and scored according to a previously published scoring system by Romano *et al*. [26].

Premedication was administered intramuscularly through injection into the biceps femoris or semitendinosus muscles. In addition, all dogs were subcutaneously administered meloxicam 0.2 mg/kg, 5 mg/mL (Melovem, Dopharma, Raamsdonksveer, Netherlands) as a pre-anesthetic.

Ten minutes after administering premedication, dogs were pre-oxygenated for 2 min before the induction agent was administered. General anesthesia was induced using IV propofol 2–4 mg/kg, 10 mg/mL (Anesia, Baxter, Utrecht, Netherlands) and midazolam 0.2 mg/kg, and 5 mg/mL (Midazolam, B. Braun, Utrecht, Netherlands) as a co-induction agent. Initially, 1 mg/kg of propofol was administered, followed by 0.2 mg/kg of midazolam, and finally, propofol was titrated until the effect, until the palpebral and swallowing reflexes were lost. Anesthesia was maintained using isoflurane 1000 mg/g (Isoflutek, Karizoo, Barcelona, Spain) in 100% oxygen after orotracheal intubation. The initial end-tidal isoflurane (EtIso) concentration was set between 1% and 1.5%, with the target EtIso of 1.1%. Ventilation was spontaneous unless apnea occurred, in which case intermittent positive pressure ventilation (10–15 breaths/min) was provided until spontaneous breathing resumed. Lactated Ringer’s solution was infused at a rate of 5 mL/kg/h during anesthesia.

Continuous lead-II electrocardiography, heart rate, respiratory rate, pulse oximetry (SpO_2_%), and non-invasive arterial blood pressure were monitored throughout the anesthesia using a pre-calibrated multiparametric monitor (BM3 Vet, GIMA, Berlin, Germany). Exhaled end-tidal carbon dioxide and isoflurane concentrations (EtIso) were assessed using a Datex-Ohmeda S/5 Anesthesia multi-purpose monitor (Helsinki, Finland). The body temperature was maintained above 37°C using electrically heated pads, blankets, and a 3M Bair–Hugher Warming Unit. In addition, the oscillometric method was used to measure arterial blood pressure. Appropriate cuffs were placed on the front leg.

The same person consistently performed medication administration and anesthesia management throughout the perioperative period.

### Perioperative IOP measurement

Baseline (T0) IOP measurements were recorded using a TonoVet rebound tonometer during the clinical examination. IOP measurements were repeated 5 (T5) and 10 min (T10) after premedication. After induction of anesthesia and intubation, the dogs were positioned in sternal recumbency, and IOP was measured immediately after intubation (Tint). The surgical procedure was initiated within 15–20 min, and IOP was measured before incision (Tbsur), after surgery (Tasur), and after extubation (Text). The dogs were transported to the post-operative ward and a kennel. The final IOP was measured before the dogs left the clinic (Tlea).

The procedures were conducted by a dedicated team who regularly operated at the University’s small animal veterinary clinic. IOP measurements were performed by the same individual: a highly experienced ophthalmologist, Professor, Doctor of Veterinary Medicine (Dr.med.vet.), and Dipl. EESVO. The equipment was regularly calibrated to ensure accuracy.

The dogs were kept under constant observation to monitor changes in cardiovascular parameters or spontaneous movements in response to stimulation during surgery. If spontaneous movements were observed, IV propofol (0.5–1.0 mg/kg) was administered, and the isoflurane concentration was raised to achieve an EtIso concentration of 1.1%–1.2%.

### Statistical analysis

All statistical analyses were performed using Microsoft Office Excel 2016 (Microsoft Office, Washington, USA). Data were assessed for normality using the Shapiro–Wilk test to determine the suitability of parametric statistical methods. A two-sample t-test (assuming equal and unequal variances) was used to compare IOP values within each group at different time points (e.g., T0 vs. Tint, T0 vs. Tasur) and between groups at corresponding time points.

Pearson’s correlation coefficient was calculated to evaluate potential relationships between IOP and demographic variables (age, sex, breed, and body weight). A correlation coefficient of >0.8 was considered strong. In addition, descriptive statistics were reported for each group, including the arithmetic mean, standard deviation (SD), and mean differences across time points.

Comparisons of IOP fluctuations across the three premedication groups were further analyzed using a one-way analysis of variance, followed by Tukey’s *post hoc* test for multiple comparisons when significant differences were detected. Statistical significance was set at p < 0.05 for all tests.

Line graphs and box plots were generated to visualize trends in IOP changes over time. SDs were examined to assess variability across different perioperative phases, particularly after intubation, where variability was expected to be higher.

## RESULTS

**Figure 1:**
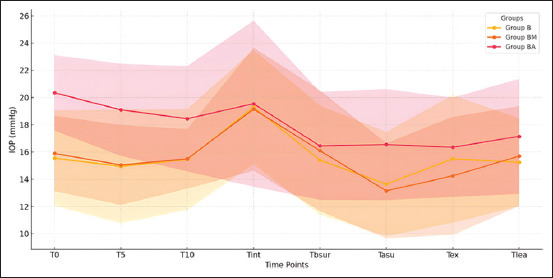
Changes in intraocular pressure (IOP) with standard deviation over the study period. B=Butorphanol only, BM=Butorphanol and medetomidine, BA=Butorphanol and acepromazine, T0=Baseline, T5=5 min after premedication, T10=10 min after premedication, Tint=After intubation, Tbsur=Before surgery, Tasur=After surgery, T_t_ext=After extubation, Tlea=Before discharge.

No significant correlations were identified between IOP and age, sex, or breed of the dogs within or among any group (p > 0.05). The lack of significant correlations between IOP and factors such as age, sex, and breed suggest that these variables may not substantially influence IOP changes during the studied procedures, reinforcing the generalizability of these findings across diverse patient populations. As shown in Table 2 and Figure 1, IOP tended to decrease after premedication, although the mean values in all groups increased following intubation. However, a significant difference between these time points was found only in groups B (p = 0.003) and BM (p = 0.01). Significant differences in IOP were observed between T0 and Tasur in the BM and BA groups, (p = 0.003) in the BM group and (p = 0.001) in the BA group, respectively. A similar but non-significant trend was observed in group B (p = 0.1). In group BA, the IOP at T0 was significantly different from that at Tlea (p = 0.007), Tbsur (p = 0.001), and Tasur (p = 0.001). It is noteworthy that the SDs are larger at certain time points, such as Tint, indicating greater variability in IOP measurements during this phase. This result could be attributed to individual physiological responses or procedural factors. In contrast, lower SDs, as observed at T0, indicate more consistent IOP measurements across the groups. Finally, no significant differences in IOP were found between healthy and operated eyes or between painful and painless procedures in any group.

**Table 2:** Perioperative intraocular pressure measurements.

Time point	Group B (mmHg, X ± SD)	Group BM (mmHg, X ± SD)	Group BA (mmHg, X ± SD)
T0	15.55 ± 3.50	15.90 ± 2.77	20.35 ± 2.78
T5	14.95 ± 4.19	15.05 ± 2.93	19.1 ± 3.39
T10	15.45 ± 3.70	15.5 ± 2.19	18.45 ± 3.87
Tint	19.3 ± 4.19	19.15 ± 4.52	19.55 ± 6.10
Tbsur	15.4 ± 4.01	16.1 ± 4.44	16.45 ± 3.97
Tasur	13.65 ± 3.83	13.15 ± 3.50	16.55 ± 4.08
Text	15.5 ± 4.68	14.25 ± 4.34	16.35 ± 3.65
Tlea	15.25 ± 3.23	15.7 ± 3.67	17.15 ± 4.22

Values are presented as the arithmetic mean (X) and standard deviation (SD). B=Butorphanol only, BM=Butorphanol and medetomidine, BA=Butorphanol and acepromazine, T0=Baseline, T5=5 min after premedication, T10=10 min after premedication, Tint=After intubation, Tbsur=Before surgery, Tasur=After surgery, Text=After extubation, Tlea=Before discharge. Manometric unit of pressure: Millimeters of mercury (mmHg)

## DISCUSSION

This study aimed to investigate the effects of three different premedication protocols (B, BM, and BA) on IOP in clinically healthy dogs undergoing mild-pain ophthalmic surgery.

### IOP measurement

Canine IOP can detect systemic and ocular diseases, making it a valuable diagnostic tool [27]. The normal range of IOP in dogs has been reported as 15–25 mmHg [28, 29]. Our recent study suggested a reference range of 14.38–16.12 mmHg for IOP in dogs, which was measured using the TonoVet [24]. The TonoVet rebound (impaction) tonometer (Tiolat Ltd., Vantaa, Finland) was used in this study [30]. TonoVet is advantageous because local anesthesia is unnecessary and is easy to use in active animals. To enable more precise measurement, the instrument has a calibration setting for dogs [30–32]. All measurements, including those obtained during surgery, were obtained in animals that were positioned sternally; therefore, neck position or tension did not influence the IOP values [33].

The central nervous system (CNS) maintains IOP within a certain range by balancing the production and outflow of aqueous humor [34], which, in turn, depends on the design and functionality of the intraocular structures involved in this process. Three main factors influence IOP, namely, scleral rigidity, external pressure, and intraocular changes. However, IOP in dogs is also influenced by other variables, such as breed, age, time of day, anesthetics and sedatives administered, measurement technique, and pressure on the head, neck, and orbital area during the assessment [32]. Since our study aimed to mimic the clinical environment and evaluate the overall effects of pre-anesthetic regimens on IOP measurements, these parameters were not examined.

### IOP and anesthesia

In conventional veterinary anesthesia, a balanced premedication protocol is used to maintain optimal intraoperative homeostasis. Appropriate management of IOP before, during, and after surgery is necessary for a successful outcome [8]. During eye surgery, low but healthy IOP is usually preferred [35] because IOP represents the ocular perfusion pressure. Elevated IOP causes ischemia and optic nerve edema by reducing blood flow to the eye. Therefore, maintaining IOP within the normal range or avoiding fluctuations throughout the perioperative period should be part of a good anesthetic regimen for ocular surgery [8]. Notably, periods of abnormal IOP were not observed in any of the three groups in our study. The drugs administered resulted in stable IOP measurements in 7 time points during the perioperative period, demonstrating their ocular efficacy and safety. Although preliminary studies have shown significant changes in IOP, many anesthetics do not cause clinically relevant changes in healthy eyes [3]. However, Paranjape *et al*. [29] suggested that anesthetic drugs may occasionally increase IOP. Hofmeister *et al*. [36] claimed that many of these studies arrived at contradictory conclusions, possibly due to drug combinations or doses, measurement timing or technology, setting, or individual animal characteristics. In our study, the mean IOP for each group was not significantly different from that at baseline after 5 or 10 min, confirming that all drugs used as premedication were appropriate and did not increase IOP.

### Premedication

Opioids are the cornerstone of effective pain management in veterinary medicine. Our results showed that butorphanol as a premedication did not cause significant changes in IOP. Measurements obtained at the 5- and 10-min time points were not significantly different from baseline measurements. Mixed opinions are still present in the literature, as some studies by Rauser et al. [18], Paranjape and Pablo [29] have reported no increase in IOP, while Douet *et al*. [12] and Blaze et al. [37] observed higher IOP values after butorphanol administration. In addition, Rauser *et al*. [18] demonstrated that butorphanol combined with dexmedetomidine or medetomidine increased IOP.

Alpha-2-adrenergic agonists are frequently used in veterinary medicine before general anesthesia to induce drowsiness and analgesia during minor surgical and diagnostic procedures [38, 39]. In addition, medetomidine and dexmedetomidine are vasoconstrictor drugs that typically have no effect on IOP [29] and are often used in combination with butorphanol [40]. Our results showed that the butorphanol-medetomidine combination had no significant effect on IOP. Measurements taken 5 and 10 min after premedication administration in the BM group showed a slight decrease in IOP compared with baseline (T0). This was expected based on the mode of action of the drugs. When butorphanol was administered with medetomidine, greater sedation and concomitant myorelaxation were achieved than following the administration of B. This is consistent with the findings of previous studies. For example, Jasien *et al*. [41] showed that dexmedetomidine can lower IOP. In addition, Chandra *et al*. [42] studied the effects of IV dexmedetomidine premedication on IOP changes and concluded that a dose of 0.4 μg/kg significantly lowered IOP approximately 5 min after delivery.

Furthermore, Artigas *et al*. [20] reported that dexmedetomidine (5 μg/kg IV) reduced IOP in 42 dogs 20 min after drug administration. Kusolphat *et al*. [1] demonstrated that an IM dexmedetomidine dose of 10 μg/kg significantly decreased IOP after 2 min. A possible explanation is that the tone of the extraocular muscles decreases with increasing sedation, limiting the increase in IOP [36]. The ability of alpha-2-adrenoceptor agonists to induce adequate sedation and myorelaxation suggests their potential to reduce IOP.

Vasodilators such as propofol tend to increase IOP [29]. In dogs premedicated with ACP (0.5 mg/kg IV) or xylazine (1 mg/kg IV), ketamine at a dose of 10 mg/kg IV caused a non-significant increase in IOP [37]. In contrast, when opioids are co-administered with ACP, they appear to modulate this increase in IOP [43, 44]. Our study showed that IOP decreased over time after premedication in group BA, similar to the findings of Tamura *et al*. [43] and Stephan *et al*. [44]. This pressure decrease can be observed at T5 and T10. The peak CNS effects of ACP typically occur 10–20 min after IV administration and 30–40 min after IM administration. The observed decrease in IOP at the time of intubation may be associated with the pharmacokinetics of ACP [14].

The mean IOP was significantly lower at Tlea, Tbsur, and Tasur than at baseline (T_0_0) after treatment with butorphanol and ACP. Slow absorption after IM administration may be associated with the onset of action of ACP [45]. Despite the temporal differences in IOP in group BA being statistically significant, the changes were minimal and are of little consequence in healthy dogs. After premedication, decreases in IOP at T5 and T10 were recorded in all three groups. However, these changes were significant only in groups B and BM until T10. These results may reflect the rapid onset of butorphanol and medetomidine. These time points may represent the absorption peaks of these two drugs when administered through infusion [45].

### Induction

Smooth induction of anesthesia with minimal or no patient discomfort is desirable. Although experimental studies have shown that propofol increases IOP, this drug is unlikely to have a clinically significant effect if administered after sufficient premedication. In a study of different propofol doses used without premedication to induce anesthesia in clinically healthy dogs, the IOP values did not differ between the groups at any time. However, the pressure before intubation was significantly higher than that measured before the induction of anesthesia [36]. Regular propofol administration in healthy dogs did not increase IOP. Although higher doses of propofol do not appear to have additional benefits in animals that cannot tolerate an abrupt increase in IOP, they may be effective in dogs that are intolerant to an abrupt rise in blood pressure during orotracheal intubation [46]. Benzodiazepines can mitigate ketamine-induced increases in IOP [36], whereas midazolam [47, 48] alone does not alter IOP. In our study, midazolam was used as a co-induction drug. Propofol was administered at various doses until the onset of action; however, none of the dogs exceeded 4 mg/kg. Sedation with hydromorphone and dexmedetomidine significantly reduces IOP in healthy dogs and may be used in animals that cannot tolerate acute increases in IOP. However, Smith *et al*. [49] reported that IOP increased significantly after both induction protocols, reversing the effects of the premedication.

Contrary findings were reported by Lehmanna *et al*. [50]; the authors discovered that co-administration of hydromorphone and dexmedetomidine at a dose of 1 μg/kg caused a statistically significant increase in IOP 30 min after drug administration. The authors claim that higher doses of dexmedetomidine are needed to decrease IOP.

Significant differences in IOP pre- and post-intubation were observed in the B and BM groups. In all three groups, temporal decreases in IOP after premedication (at T5 and T10) were observed, although the mean values were elevated following intubation, likely due to increased sympathetic nervous system stimulation after endotracheal intubation [45]. Due to sympathetic stimulation following laryngoscopy and endotracheal intubation in humans, an increase in IOP above pre-intubation levels has been reported by Ismail *et al*. [51]; however, administration of dexmedetomidine as a pre-anesthetic reduced this pressure response [42]. Chandra *et al*. [42] reported that premedication with dexmedetomidine reduced the increase in IOP caused by dissociative drugs, laryngoscopy, and intubation. However, our study observed a significant difference in IOP between baseline and post-intubation. This result can be explained by the lower medetomidine dose used (0.005 mg/kg) compared with that used in previous studies by Mrazova *et al*. [13], Aghababaei *et al*. [16], and Chandra *et al*. [42].

In the BA group, IOP was significantly lower at Tlea, Tbsur, and Tasur than at T0. This may be due to the peak ACP concentration. When ACP and butorphanol were combined, the onset and duration of action were longer in the BM and B groups. This suggests that ACP is systemically absorbed more slowly than medetomidine. Although differences were found at Tlea, Tbsur, and Tasur, these were minimal and were of little significance in healthy dogs. The significant differences observed in the BA group between T0 and subsequent time points (Tlea, Tbsur, and Tasur) may reflect the pharmacodynamic effects of the premedication used, particularly in maintaining intraocular stability during surgical procedures. This highlights the potential importance of tailored anesthetic protocols in managing patients with elevated IOP, for instance, breed-related glaucoma patients: American Cocker Spaniel, Basset Hound, Chow Chow, and Shar-Pei [52]. Measurements obtained after extubation and before discharge were similar in all three groups and did not exceed baseline values.

Overall, our results corroborate Dewangan’s outcomes [53] and support the use of butorphanol in mild-pain ophthalmic surgery due to its pharmacological effects that reduce cough stimulus and increase IOP [45, 54].

Neither the differences in IOP between painless and painful surgeries nor those between healthy and operated eyes were statistically significant in any group. Since our study focused on ophthalmic surgeries classified as mildly painful, and each animal received analgesics beforehand, variation in nociception is unlikely to have affected IOP.

## Limitations

The current clinical trial has several limitations. The simultaneous administration of additional anesthetic agents for the induction and maintenance of general anesthesia may have affected IOP. Furthermore, differences in the breed, size, and temperament of the animals may have influenced the results. In the perioperative period, we were not able to assess the IOP during the painful procedure. Dogs predisposed to breed-related glaucoma may exhibit higher IOP compared to healthy individuals without this predisposition.

The results of the study may be limited by the fact that no parallel studies of other teams using identical methods have been conducted, which could limit the generalizability of the results with different training, skill levels, and procedural preferences. There may also be an experience bias because the results reflect the expertise and practices of a single team and may overlook differences in other clinical settings. To improve generalizability, future studies should include multiple teams or centers and larger samples to capture different methods and improve statistical power.

## CONCLUSION

This study evaluated the effects of three different premedication protocols on IOP dynamics in dogs undergoing mild-pain ophthalmic procedures. The results demonstrated that all three protocols – B, BM, and BA – maintained IOP within the normal physiological range throughout the perioperative period. However, notable differences were observed among the groups, particularly in response to intubation and surgical stimulation. The BA protocol (butorphanol + ACP) was the most effective in attenuating IOP spikes associated with intubation, suggesting its potential benefit in patients at risk of corneal perforation or elevated IOP. The B and BM groups exhibited transient but significant IOP elevations after intubation, reinforcing the need for careful anesthetic selection in ophthalmic procedures. No significant correlation was found between IOP and sex, breed, age, or body weight, indicating that these factors do not substantially influence IOP changes in healthy dogs undergoing mild ophthalmic surgery. In addition, the lack of significant differences in IOP between healthy and operated eyes suggests that the premedication protocols had a more pronounced effect on IOP regulation than the surgical procedure itself. For patients at risk of corneal perforation or increased IOP (e.g., breeds predisposed to glaucoma such as Cocker Spaniels, Chow Chows, and Basset Hounds), a combination of butorphanol and ACP (BA protocol) is recommended to minimize IOP fluctuations and maintain ocular stability. B protocol or BM protocol should be used cautiously in cases where IOP control is critical, as both were associated with transient but significant increases in IOP after intubation. In high-risk ophthalmic surgeries, such as those involving deep corneal ulcers or lens instability, premedication with ACP may be preferable to medetomidine, given its more consistent IOP-lowering effects. Close IOP monitoring is essential, especially during intubation and extubation, as these are critical points where IOP fluctuations may occur. Additional intraoperative interventions, such as smooth induction and analgesic management, should be considered to maintain IOP stability. Further studies are needed to evaluate the effects of these protocols in dogs with pre-existing ocular conditions, particularly those with glaucoma, chronic uveitis, or previous ophthalmic surgery, to optimize individualized anesthetic protocols for these patients. From a clinical perspective, selecting an appropriate premedication strategy is crucial for optimizing ocular perfusion, reducing intraoperative fluctuations, and minimizing potential complications. The findings suggest that ACP in combination with butorphanol is a preferable choice for patients at risk of IOP elevation. Future studies should investigate these effects in dogs with pre-existing ocular conditions, particularly those prone to glaucoma or corneal pathology, to enhance clinical decision-making for veterinary ophthalmic anesthesia. These results provide valuable insights into perioperative IOP management, reinforcing the importance of tailored anesthetic protocols to improve surgical outcomes and animal welfare in veterinary ophthalmology.

## AUTHORS’ CONTRIBUTIONS

LK and LV: Conceptualization and methodology and drafted and revised the manuscript. LK, LV, and DB: Collected clinical data and performed IOP measurements. AV: Data analysis and drafted and revised the manuscript. All authors have read and approved the final manuscript.
